# Supplementary material for: Public perception of scientists: Experimental evidence on the role of sociodemographic, partisan, and professional characteristics
Source: PLoS One. 2023 Jul 7;18(7):e0287572. doi: 10.1371/journal.pone.0287572 (PMC10328236; doi:10.1371/journal.pone.0287572)
Supplement: S1 Appendix — (ZIP) [file pone.0287572.s001.zip › S1 Appendix.docx]

S1 Appendix: Public perception of scientists: Experimental evidence on the role of sociodemographic, partisan, and professional characteristics

**S1) Survey Experiment and Sample Characteristics**

Our survey experiment was fielded in the United States in March 2020. We recruited 1005 participants through Prolific Academic, a large online panel with over 40,000 active participants. Based on Prolific’s representative quota sampling, [Figure](#figures2) 1 shows that our sample is fairly representative of the U.S. population in terms of age, sex, and ethnicity.

Our empirical strategy is based on a conjoint survey experiment. This experimental technique allows us to identify individuals’ perception of scientists, simultaneously manipulating multiple attributes of scientists through different profiles [1]. This experimental design provides research subjects with paired profiles of scientists, whose attributes are randomly varied, to ask their preferences over the profiles. In doing so, we are able to identify the causal impact of each attribute of scientists over research subjects’ preference for a certain profile. Employing the conjoint experiment, we also aim to better unveil attitudes on sensitive questions such as the effect of scientist’s sex or race on public preferences, since this experimental design allows respondents to justify any particular preference with a number of reasons [2].

In our conjoint design, we created a scenario where respondents are asked to compare and judge the profiles of hypothetical scientists, in five rounds, who vary along multiple dimensions, such as sex, race/ethnicity, scientific field, place of work, and in some randomly selected cases, political party identification. In addition, in our design, respondents also rate the profiles of scientists on a scale based on different dimensions of trust. [Figure 1](#figures1) illustrates the instructions and paired conjoint profiles as seen by respondents during the study.


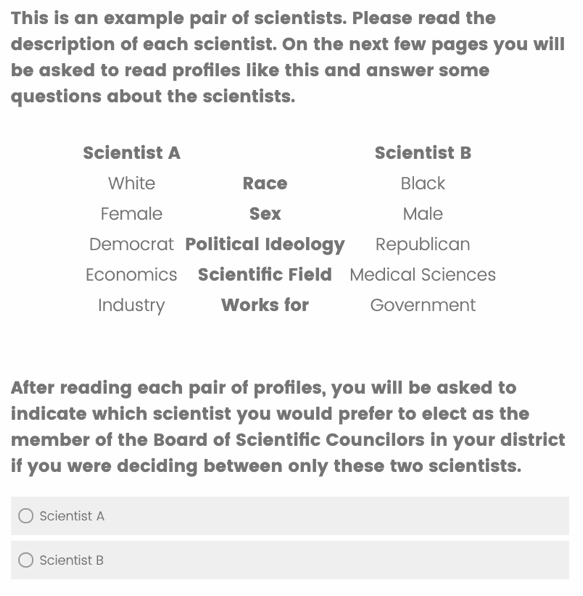


1. Instructional page


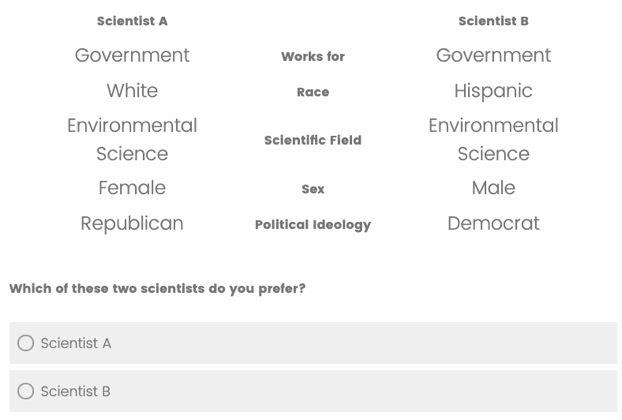


1. An example of paired profiles

Figure 1. Conjoint Profiles


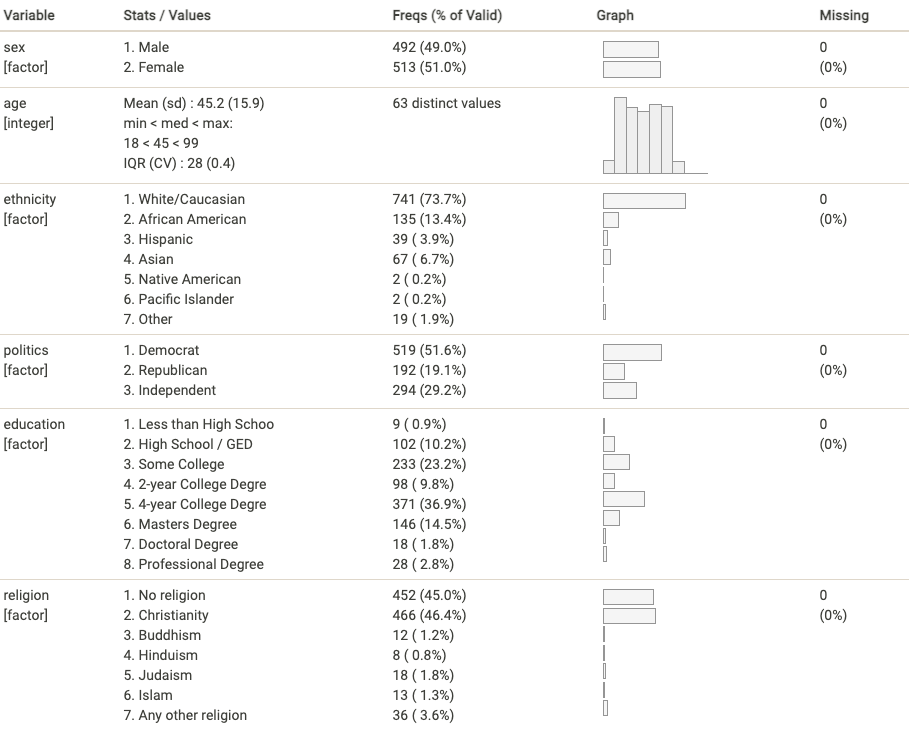


Figure 2: Sample Characteristics

**S2) Covariate Balance Testing and Carryover Effect Testing**

[Table 1](#tables1) and [Figure 3](#figures3) show that the randomization of receiving partisan prompt in a paired profiles conjoint balanced potential confounding factors on average. This ensures the internal validity of the between-subjects design by conducting the Kolmogorov–Smirnov and Chi-squared tests for covariate balance check below.

| **Covariate** | **Not receiving partisan prompt (n=502)** | **Receiving partisan prompt (n=503)** |  |  |
| --- | --- | --- | --- | --- |
| **Age** | | |  |  |
| Age | 45.37 | 45.00 |  |  |
| **Sex** | | |  |  |
| Male | 0.46 | 0.51 |  |  |
| Female | 0.54 | 0.49 |  |  |
| **Ethnicity** | | |  |  |
| White/Caucasian | 0.73 | 0.74 |  |  |
| African American | 0.14 | 0.13 |  |  |
| Hispanic | 0.05 | 0.03 |  |  |
| Asian | 0.06 | 0.08 |  |  |
| Native American | 0.00 | 0.00 |  |  |
| Pacific Islander | 0.00 | 0.00 |  |  |
| Other | 0.02 | 0.02 |  |  |
| **Party Affiliation** | | |  |  |
| Democrat | 0.52 | 0.51 |  |  |
| Republican | 0.19 | 0.19 |  |  |
| Independent | 0.29 | 0.29 |  |  |
| **Education** | | |  |  |
| Less than High School | 0.01 | 0.01 |  |  |
| High School / GED | 0.11 | 0.09 |  |  |
| Some College | 0.22 | 0.24 |  |  |
| 2-year College Degree | 0.11 | 0.09 |  |  |
| 4-year College Degree | 0.39 | 0.35 |  |  |
| Masters Degree | 0.13 | 0.16 |  |  |
| Doctoral Degree | 0.02 | 0.02 |  |  |
| Professional Degree (JD, MD) | 0.02 | 0.03 |  |  |
| **Religiosity** | | |  |  |
| No religion | 0.46 | 0.44 |  |  |
| Christianity | 0.46 | 0.47 |  |  |
| Buddhism | 0.01 | 0.01 |  |  |
| Hinduism | 0.01 | 0.01 |  |  |
| Judaism | 0.01 | 0.03 |  |  |
| Islam | 0.01 | 0.01 |  |  |
| Any other religion | 0.04 | 0.03 |  |  |

Table 1: Covariate Balance Check

We also controlled whether our within-subjects design is balanced. In order to ensure that the each level of attributes is uniformly distributed, we compared a covariate (respondent’s age) across different levels of attributes. Confidence intervals of marginal means in [Figure 4](#figures4) show that the potential imbalance does not significantly affect our estimates. In ensuring the internal validity of within-subjects design in our conjoint experiment, we also checked the assumption that respondents do not assess the profiles in subsequent rounds with carrying over the effect from one task to another so that multiple observations from the same respondent can be treated as independent of one another. [Figure 5](#figures5) shows that there is no considerable pattern for such instances.


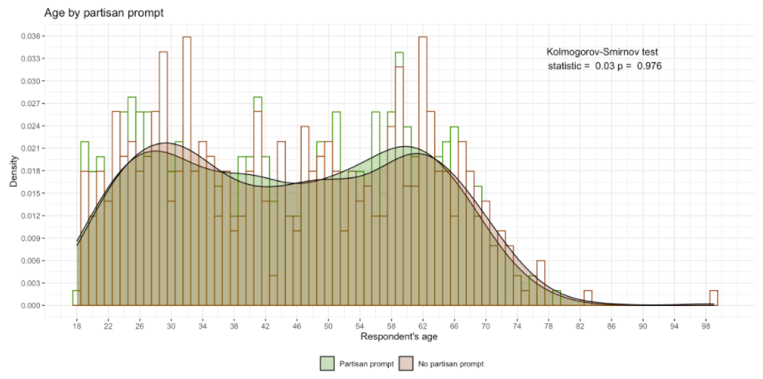


(a) Age by Partisanship


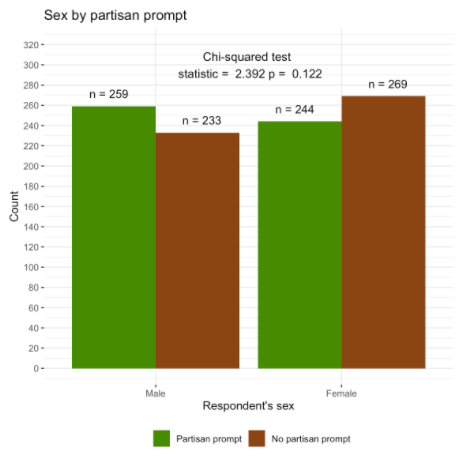


(b) Sex by Partisanship


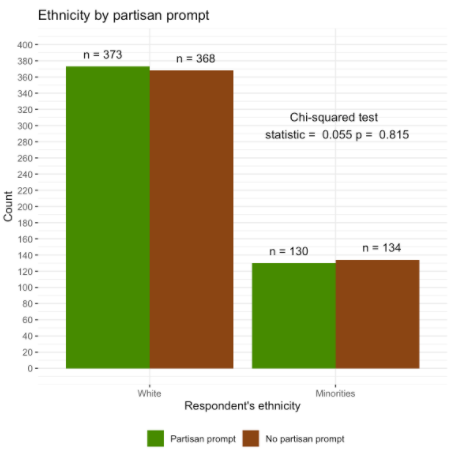


(c) Ethnicity by Partisanship


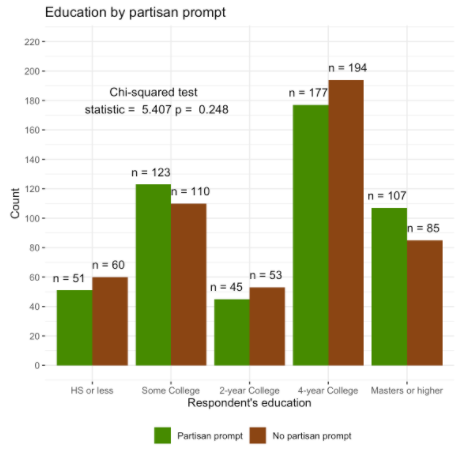


(d) Education by Partisanship


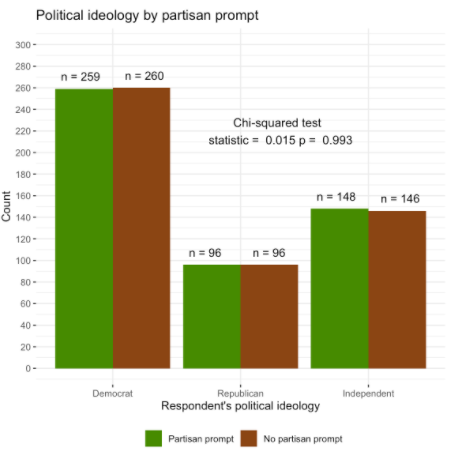


(e) Political Identification by Partisanship


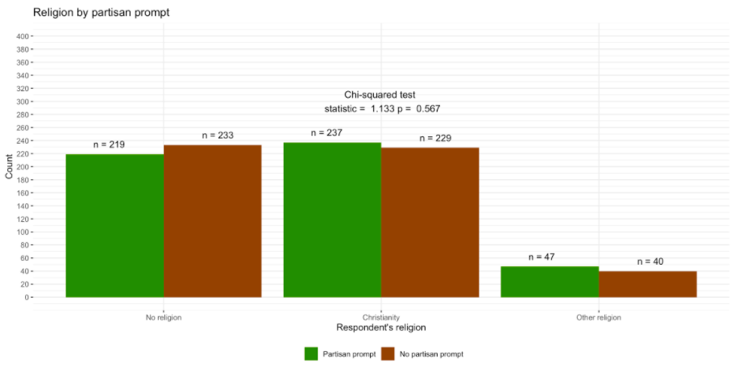


(f) Religion by Partisanship

Figure 3. Covariate Balance


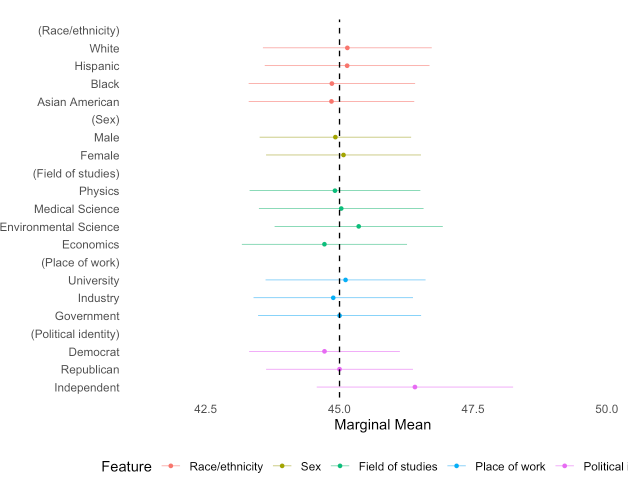


1. Balance testing using respondent’s age for profiles with partisan prompt


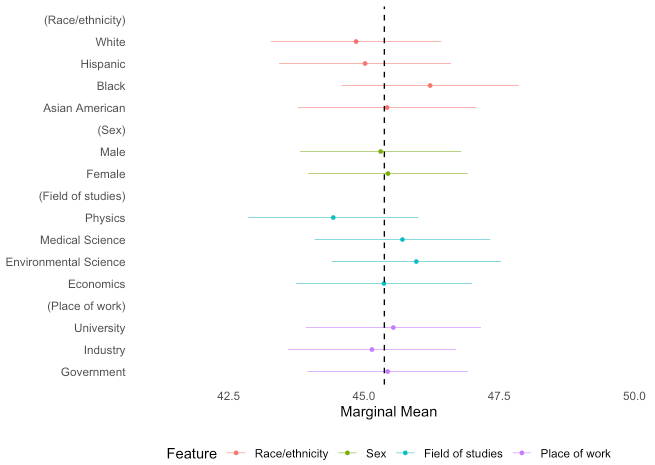


1. Balance testing using respondent’s age for profiles without partisan prompt

Figure 4. Balance Testing


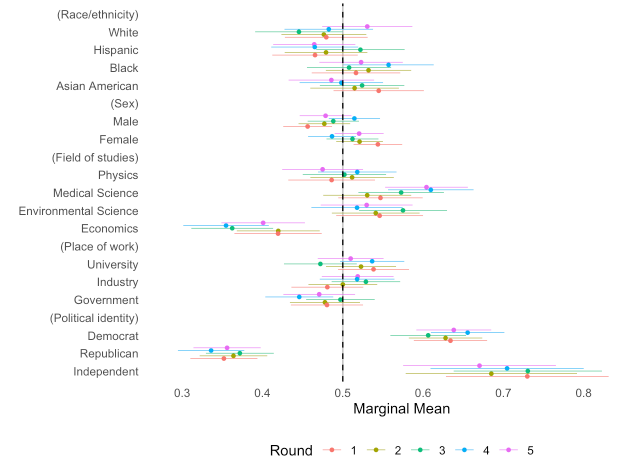


1. Profiles with partisan prompt


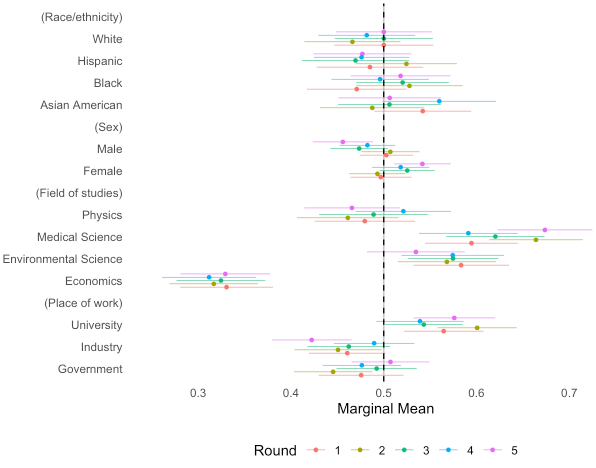


1. Profiles without partisan prompt

Figure 5. Testing Carryover Effects

**S3) Analysis with Entropy Balancing**

As a further robustness check, we tested that there were no other biases in our online quota sample that was representative of the U.S. population based on key demographic variables, such as age, sex, and ethnicity but politically skewed to Democrats. In order to ensure representativeness, we first implemented entropy balancing to weight our sample in terms of quota indicators: age, sex, ethnicity, and then partisan characteristics. This method adjusts differences in the first, second, and third moment of the covariate distributions, such as covariate means, variances, and skewness (for a detailed discussion, see Hainmueller and Xu (2013)) [3]. [Tables 11](#tables11),[12](#tables12) and [Figure 6](#figures6) illustrate that we do not observe any major differences in the preferences for scientists when we adjust our sample to both quota and partisan characteristics.


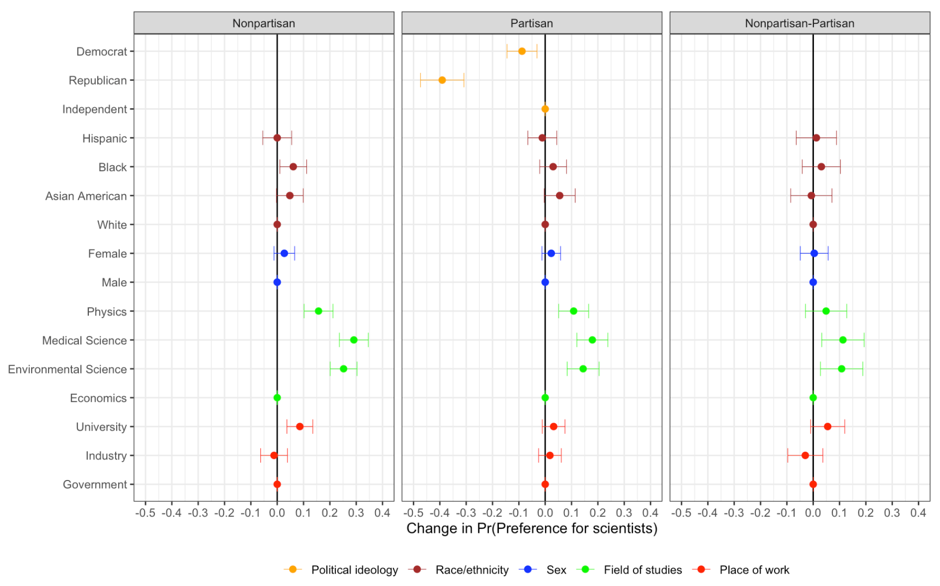
a) Estimated Average Marginal Component Effects with Quota-adjusted Weights (Age, Sex, Ethnicity)


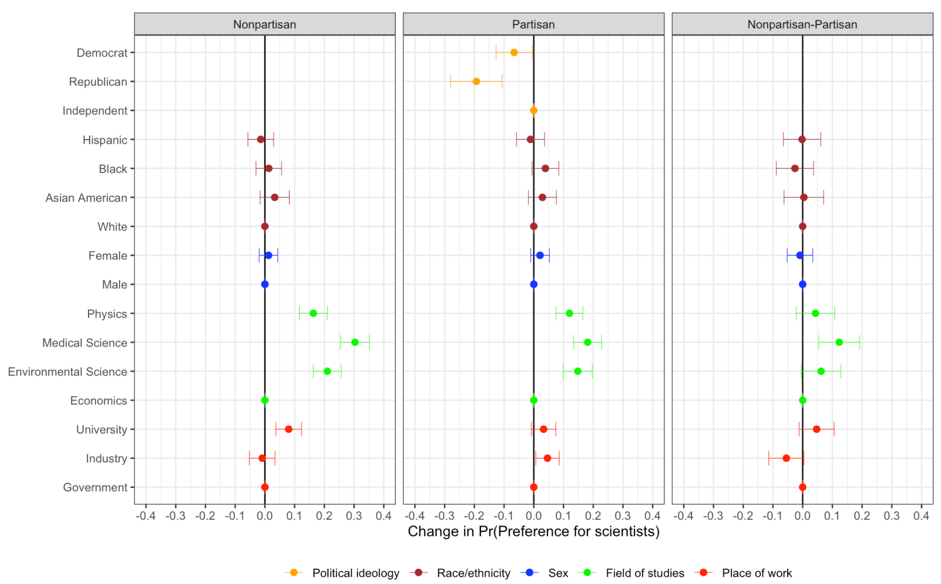


b) Estimated Average Marginal Component Effects with Partisanship-adjusted Weights

Figure 6. Weighted Results

**S4) Regression Outputs**

Table 3: The Main Model – Aggregate Results

|  | **(Non-partisan N=5020)** | | | | **(Partisan N=5030)** | | | | **(Difference N=10050)** | | | |
| --- | --- | --- | --- | --- | --- | --- | --- | --- | --- | --- | --- | --- |
|  | **Est.** | **SE** | **Stat.** | **P-Value** | **Est.** | **SE** | **Stat.** | **P-Value** | **Est.** | **SE** | **Stat.** | **P-Value** |
| Intercept | 0.268 | 0.022 | 12.348 | < 0.001 | 0.534 | 0.034 | 15.630 | <0.001 | 0.534 | 0.034 | 15.638 | <0.001 |
| Democrat | NA | NA | NA | NA | -0.073 | 0.025 | -2.979 | 0.003 | NA | NA | NA | NA |
| Republican | NA | NA | NA | NA | -0.350 | 0.035 | -10.099 | <0.001 | NA | NA | NA | NA |
| Female | 0.030 | 0.015 | 2.010 | 0.045 | 0.033 | 0.014 | 2.422 | 0.015 | -0.003 | 0.020 | -0.168 | 0.867 |
| Asian American | 0.039 | 0.020 | 1.895 | 0.058 | 0.038 | 0.019 | 1.959 | 0.050 | 0.001 | 0.028 | 0.025 | 0.980 |
| Black | 0.026 | 0.020 | 1.320 | 0.187 | 0.046 | 0.020 | 2.323 | 0.020 | -0.020 | 0.028 | -0.706 | 0.480 |
| Hispanic | 0.003 | 0.020 | 0.161 | 0.872 | -0.003 | 0.020 | -0.138 | 0.890 | 0.006 | 0.028 | 0.211 | 0.833 |
| Env. Science | 0.245 | 0.021 | 11.616 | <0.001 | 0.153 | 0.021 | 7.118 | <0.001 | 0.092 | 0.030 | 3.061 | 0.002 |
| Medical Science | 0.308 | 0.021 | 14.413 | <0.001 | 0.183 | 0.021 | 8.601 | <0.001 | 0.124 | 0.030 | 4.122 | 0.000 |
| Physics | 0.163 | 0.021 | 7.758 | <0.001 | 0.114 | 0.020 | 5.683 | <0.001 | 0.049 | 0.029 | 1.668 | 0.095 |
| Industry | -0.021 | 0.019 | -1.068 | 0.286 | 0.034 | 0.017 | 1.983 | 0.047 | -0.055 | 0.026 | -2.111 | 0.035 |
| University | 0.088 | 0.018 | 4.760 | <0.001 | 0.033 | 0.017 | 1.909 | 0.056 | 0.055 | 0.025 | 2.168 | 0.030 |

Notes: Standard errors clustered at respondent level. Reference categories are: Independent, White, Male, Economics, Government

Table 4: Heterogeneous Treatment Effects by R’s Party Identification - Non-partisan Model

|  | **(Democrats N= 2600)** | | | | **(Republicans N=960)** | | | | |
| --- | --- | --- | --- | --- | --- | --- | --- | --- | --- |
|  | **Est.** | **SE** | **Stat.** | **P-Value** | **Est.** | **SE** | **Stat.** | **P-Value** |  |
| Intercept | 0.246 | 0.03 | 8.175 | <0.001 | 0.38 | 0.049 | 7.817 | <0.001 |  |
| Female | 0.077 | 0.021 | 3.611 | <0.001 | -0.062 | 0.029 | -2.162 | 0.031 |  |
| Asian American | 0.015 | 0.028 | 0.532 | 0.595 | 0.031 | 0.051 | 0.598 | 0.55 |  |
| Black | 0.041 | 0.027 | 1.511 | 0.131 | -0.029 | 0.045 | -0.652 | 0.515 |  |
| Hispanic | 0.02 | 0.028 | 0.729 | 0.466 | -0.067 | 0.041 | -1.646 | 0.1 |  |
| Environmental Science | 0.279 | 0.03 | 9.329 | <0.001 | 0.106 | 0.047 | 2.267 | 0.024 |  |
| Medical Science | 0.3 | 0.03 | 9.94 | <0.001 | 0.301 | 0.051 | 5.862 | <0.001 |  |
| Physics | 0.139 | 0.029 | 4.759 | <0.001 | 0.174 | 0.048 | 3.602 | <0.001 |  |
| Industry | -0.037 | 0.027 | -1.39 | 0.165 | 0.018 | 0.045 | 0.396 | 0.692 |  |
| University | 0.095 | 0.026 | 3.703 | <0.001 | 0.049 | 0.043 | 1.144 | 0.253 |  |

Notes: Standard errors clustered at respondent level. Reference categories are: White, Male, Economics, Government

Table 5: Heterogeneous Treatment Effects by R’s Party Identification – Partisan Model

|  | **(Democrats N= 2590)** | | | | **(Republicans N=960)** | | | | |
| --- | --- | --- | --- | --- | --- | --- | --- | --- | --- |
|  | **Est.** | **SE** | **Stat.** | **P-Value** | **Est.** | **SE** | **Stat.** | **P-Value** |  |
| Intercept | 0.677 | 0.036 | 18.734 | <0.001 | 0.21 | 0.08 | 2.636 | 0.009 |  |
| Democrat | -0.056 | 0.025 | -2.222 | 0.026 | -0.086 | 0.057 | -1.519 | 0.129 |  |
| Republican | -0.671 | 0.033 | -20.035 | <0.001 | 0.38 | 0.069 | 5.508 | <0.001 |  |
| Female | 0.03 | 0.016 | 1.912 | 0.056 | -0.018 | 0.032 | -0.568 | 0.57 |  |
| Asian American | 0.07 | 0.021 | 3.367 | 0.001 | 0.005 | 0.042 | 0.125 | 0.9 |  |
| Black | 0.039 | 0.02 | 1.96 | 0.05 | 0.009 | 0.039 | 0.231 | 0.817 |  |
| Hispanic | 0.019 | 0.022 | 0.862 | 0.389 | -0.018 | 0.044 | -0.396 | 0.692 |  |
| Environmental Science | 0.183 | 0.027 | 6.805 | <0.001 | 0.105 | 0.045 | 2.303 | 0.021 |  |
| Medical Science | 0.173 | 0.024 | 7.129 | <0.001 | 0.188 | 0.047 | 4.034 | <0.001 |  |
| Physics | 0.108 | 0.022 | 4.849 | <0.001 | 0.145 | 0.046 | 3.133 | 0.002 |  |
| Industry | 0.003 | 0.020 | 0.154 | 0.877 | 0.082 | 0.037 | 2.197 | 0.028 |  |
| University | 0.058 | 0.020 | 2.859 | 0.004 | 0.027 | 0.038 | 0.713 | 0.476 |  |

Notes: Standard errors clustered at respondent level. Reference categories are: Independent, White, Male, Economics, Government

Table 6: Heterogeneous Treatment Effects by R’s Party Identification –

Non-partisan-Partisan Difference Model

|  | **(Democrats N= 5190)** | | | | **(Republicans N=1920)** | | | | |
| --- | --- | --- | --- | --- | --- | --- | --- | --- | --- |
|  | **Est.** | **SE** | **Stat.** | **P-Value** | **Est.** | **SE** | **Stat.** | **P-Value** |  |
| Intercept | 0.677 | 0.036 | 18.754 | <0.001 | 0.210 | 0.080 | 2.644 | 0.008 |  |
| Female | 0.046 | 0.026 | 1.742 | 0.082 | -0.044 | 0.043 | -1.030 | 0.303 |  |
| Asian American | -0.055 | 0.035 | -1.605 | 0.109 | 0.025 | 0.066 | 0.385 | 0.700 |  |
| Black | 0.001 | 0.034 | 0.041 | 0.967 | -0.038 | 0.059 | -0.643 | 0.521 |  |
| Hispanic | 0.001 | 0.035 | 0.036 | 0.971 | -0.049 | 0.060 | -0.823 | 0.411 |  |
| Environmental Science | 0.096 | 0.040 | 2.386 | 0.017 | 0.002 | 0.065 | 0.026 | 0.979 |  |
| Medical Science | 0.127 | 0.039 | 3.267 | 0.001 | 0.112 | 0.069 | 1.620 | 0.105 |  |
| Physics | 0.031 | 0.037 | 0.859 | 0.391 | 0.029 | 0.067 | 0.432 | 0.666 |  |
| Industry | -0.041 | 0.034 | -1.207 | 0.228 | -0.064 | 0.058 | -1.102 | 0.270 |  |
| University | 0.038 | 0.033 | 1.149 | 0.251 | 0.023 | 0.057 | 0.397 | 0.692 |  |

Notes: Standard errors clustered at respondent level. Reference categories are: White, Male, Economics, Government

Table 7: The Main Model – Results with Particular Trust

|  | **(Non-partisan N=5020)** | | | | **(Partisan N=5030)** | | | | **(Difference N=10050)** | | | |
| --- | --- | --- | --- | --- | --- | --- | --- | --- | --- | --- | --- | --- |
|  | **Est.** | **SE** | **Stat.** | **P-Value** | **Est.** | **SE** | **Stat.** | **P-Value** | **Est.** | **SE** | **Stat.** | **P-Value** |
| Intercept | 0.734 | 0.024 | 30.607 | <0.001 | 0.751 | 0.029 | 25.689 | <0.001 | 0.751 | 0.029 | 25.703 | <0.001 |
| Democrat | NA | NA | NA | NA | -0.077 | 0.021 | -3.661 | <0.001 | NA | NA | NA | NA |
| Republican | NA | NA | NA | NA | -0.181 | 0.023 | -7.883 | <0.001 | NA | NA | NA | NA |
| Female | -0.001 | 0.012 | -0.109 | 0.913 | -0.010 | 0.012 | -0.865 | 0.387 | 0.009 | 0.017 | 0.512 | 0.608 |
| Asian American | 0.010 | 0.015 | 0.686 | 0.492 | 0.047 | 0.018 | 2.584 | 0.010 | -0.036 | 0.024 | -1.547 | 0.122 |
| Black | 0.026 | 0.015 | 1.690 | 0.091 | 0.030 | 0.018 | 1.642 | 0.101 | -0.005 | 0.024 | -0.197 | 0.843 |
| Hispanic | 0.024 | 0.015 | 1.618 | 0.106 | 0.036 | 0.018 | 1.988 | 0.047 | -0.011 | 0.023 | -0.478 | 0.632 |
| Env. Science | 0.061 | 0.019 | 3.266 | 0.001 | 0.057 | 0.018 | 3.122 | 0.002 | 0.004 | 0.026 | 0.134 | 0.894 |
| Medical Science | 0.082 | 0.019 | 4.346 | <0.001 | 0.076 | 0.020 | 3.830 | <0.001 | 0.006 | 0.027 | 0.214 | 0.831 |
| Physics | 0.046 | 0.019 | 2.419 | 0.016 | 0.071 | 0.019 | 3.730 | <0.001 | -0.025 | 0.027 | -0.937 | 0.349 |
| Industry | -0.021 | 0.018 | -1.188 | 0.235 | 0.013 | 0.016 | 0.827 | 0.408 | -0.034 | 0.024 | -1.437 | 0.151 |
| University | 0.040 | 0.016 | 2.566 | 0.010 | 0.047 | 0.016 | 2.931 | 0.003 | -0.006 | 0.022 | -0.290 | 0.772 |

Notes: Standard errors clustered at respondent level. Reference categories are: Independent, White, Male, Economics, Government

Table 8: The Main Model – Results with Epistemic Trustworthiness

|  | **(Non-partisan N=5020)** | | | | **(Partisan N=5030)** | | | | **(Difference N=10050)** | | | |
| --- | --- | --- | --- | --- | --- | --- | --- | --- | --- | --- | --- | --- |
|  | **Est.** | **SE** | **Stat.** | **P-Value** | **Est.** | **SE** | **Stat.** | **P-Value** | **Est.** | **SE** | **Stat.** | **P-Value** |
| Intercept | 0.809 | 0.021 | 38.935 | <0.001 | 0.796 | 0.025 | 32.118 | <0.001 | 0.796 | 0.025 | 32.135 | <0.001 |
| Democrat | NA | NA | NA | NA | -0.042 | 0.016 | -2.559 | 0.011 | NA | NA | NA | NA |
| Republican | NA | NA | NA | NA | -0.063 | 0.017 | -3.626 | <0.001 | NA | NA | NA | NA |
| Female | -0.001 | 0.009 | -0.141 | 0.888 | 0.003 | 0.010 | 0.316 | 0.752 | -0.004 | 0.013 | -0.329 | 0.742 |
| Asian American | -0.002 | 0.012 | -0.185 | 0.854 | 0.001 | 0.015 | 0.051 | 0.960 | -0.003 | 0.019 | -0.156 | 0.876 |
| Black | 0.001 | 0.014 | 0.046 | 0.963 | 0.024 | 0.014 | 1.670 | 0.095 | -0.023 | 0.020 | -1.156 | 0.248 |
| Hispanic | 0.014 | 0.013 | 1.136 | 0.256 | -0.004 | 0.014 | -0.265 | 0.791 | 0.018 | 0.019 | 0.952 | 0.341 |
| Env. Science | 0.081 | 0.018 | 4.365 | <0.001 | 0.124 | 0.018 | 6.779 | <0.001 | -0.043 | 0.026 | -1.662 | 0.096 |
| Medical Science | 0.108 | 0.018 | 6.185 | <0.001 | 0.144 | 0.018 | 7.850 | 0.000 | -0.035 | 0.025 | -1.400 | 0.162 |
| Physics | 0.065 | 0.019 | 3.494 | <0.001 | 0.123 | 0.019 | 6.544 | <0.001 | -0.058 | 0.026 | -2.211 | 0.027 |
| Industry | -0.003 | 0.012 | -0.249 | 0.803 | <0.001 | 0.013 | 0.016 | 0.988 | -0.003 | 0.018 | -0.182 | 0.856 |
| University | 0.022 | 0.012 | 1.839 | 0.066 | 0.007 | 0.012 | 0.600 | 0.548 | 0.015 | 0.017 | 0.860 | 0.390 |

Notes: Standard errors clustered at respondent level. Reference categories are: Independent, White, Male, Economics, Government

Table 9: The Main Model – Results with Normative Trustworthiness

|  | **(Non-partisan N=5020)** | | | | **(Partisan N=5030)** | | | | **(Difference N=10050)** | | | |
| --- | --- | --- | --- | --- | --- | --- | --- | --- | --- | --- | --- | --- |
|  | **Est.** | **SE** | **Stat.** | **P-Value** | **Est.** | **SE** | **Stat.** | **P-Value** | **Est.** | **SE** | **Stat.** | **P-Value** |
| Intercept | 0.768 | 0.023 | 34.070 | <0.001 | 0.795 | 0.027 | 29.756 | <0.001 | 0.795 | 0.027 | 29.772 | <0.001 |
| Democrat | NA | NA | NA | NA | -0.074 | 0.019 | -3.847 | <0.001 | NA | NA | NA | NA |
| Republican | NA | NA | NA | NA | -0.183 | 0.021 | -8.561 | <0.001 | NA | NA | NA | NA |
| Female | 0.010 | 0.012 | 0.822 | 0.411 | 0.005 | 0.012 | 0.414 | 0.679 | 0.005 | 0.017 | 0.293 | 0.770 |
| Asian American | 0.011 | 0.014 | 0.791 | 0.429 | 0.031 | 0.018 | 1.736 | 0.083 | -0.020 | 0.023 | -0.857 | 0.391 |
| Black | 0.002 | 0.015 | 0.164 | 0.870 | 0.026 | 0.018 | 1.403 | 0.161 | -0.024 | 0.024 | -0.994 | 0.320 |
| Hispanic | 0.017 | 0.014 | 1.173 | 0.241 | 0.041 | 0.017 | 2.356 | 0.019 | -0.024 | 0.023 | -1.077 | 0.281 |
| Env. Science | 0.076 | 0.019 | 4.074 | <0.001 | 0.066 | 0.019 | 3.539 | <0.001 | 0.010 | 0.026 | 0.368 | 0.713 |
| Medical Science | 0.096 | 0.018 | 5.463 | <0.001 | 0.059 | 0.019 | 3.114 | 0.002 | 0.037 | 0.026 | 1.429 | 0.153 |
| Physics | 0.032 | 0.018 | 1.748 | 0.081 | 0.050 | 0.019 | 2.675 | 0.007 | -0.018 | 0.026 | -0.685 | 0.493 |
| Industry | -0.043 | 0.018 | -2.475 | 0.013 | -0.021 | 0.015 | -1.403 | 0.161 | -0.022 | 0.023 | -0.952 | 0.341 |
| University | 0.035 | 0.014 | 2.455 | 0.014 | 0.021 | 0.014 | 1.434 | 0.152 | 0.015 | 0.020 | 0.725 | 0.469 |

Notes: Standard errors clustered at respondent level. Reference categories are: Independent, White, Male, Economics, Government

Table 10: The Interaction Model – Conditional on Scientist’s Party Identification

| Political ideology | Place of work | Estimate | Std. Error | Statistic | P-Value |
| --- | --- | --- | --- | --- | --- |
| Independent | Government | NA | NA | NA | NA |
| Independent | Industry | 0.047 | 0.056 | 0.842 | 0.400 |
| Independent | University | 0.027 | 0.054 | 0.490 | 0.624 |
| Republican | Government | NA | NA | NA | NA |
| Republican | Industry | 0.008 | 0.024 | 0.346 | 0.730 |
| Republican | University | 0.036 | 0.025 | 1.473 | 0.141 |
| Democrat | Government | NA | NA | NA | NA |
| Democrat | Industry | 0.057 | 0.026 | 2.208 | 0.027 |
| Democrat | University | 0.034 | 0.027 | 1.270 | 0.204 |
| Democrat - Independent | Industry | 0.010 | 0.061 | 0.157 | 0.875 |
| Republican - Independent | Industry | -0.039 | 0.060 | -0.646 | 0.518 |
| Democrat - Independent | University | 0.007 | 0.061 | 0.120 | 0.904 |
| Republican - Independent | University | 0.010 | 0.060 | 0.164 | 0.870 |

Notes: Standard errors clustered at respondent level.

Table 11: The Main results with Quota-adjusted Entropy Weights

|  | **(Non-partisan N=5020)** | | | | **(Partisan N=5030)** | | | | **(Difference N=10050)** | | | |
| --- | --- | --- | --- | --- | --- | --- | --- | --- | --- | --- | --- | --- |
|  | **Est.** | **SE** | **Stat.** | **P-Value** | **Est.** | **SE** | **Stat.** | **P-Value** | **Est.** | **SE** | **Stat.** | **P-Value** |
| Intercept | 0.258 | 0.026 | 10.070 | <0.001 | 0.578 | 0.043 | 13.578 | <0.001 | 0.578 | 0.043 | 13.586 | <0.001 |
| Democrat | NA | NA | NA | NA | -0.088 | 0.029 | -3.058 | 0.002 | NA | NA | NA | NA |
| Republican | NA | NA | NA | NA | -0.391 | 0.042 | -9.287 | 0.000 | NA | NA | NA | NA |
| Female | 0.027 | 0.020 | 1.334 | 0.182 | 0.023 | 0.018 | 1.250 | 0.211 | 0.004 | 0.027 | 0.155 | 0.877 |
| Asian American | 0.048 | 0.026 | 1.837 | 0.066 | 0.055 | 0.030 | 1.861 | 0.063 | -0.007 | 0.040 | -0.180 | 0.857 |
| Black | 0.061 | 0.026 | 2.350 | 0.019 | 0.030 | 0.026 | 1.133 | 0.257 | 0.031 | 0.037 | 0.832 | 0.405 |
| Hispanic | 0.000 | 0.028 | 0.014 | 0.989 | -0.011 | 0.028 | -0.412 | 0.680 | 0.012 | 0.039 | 0.301 | 0.764 |
| Env. Science | 0.252 | 0.026 | 9.633 | <0.001 | 0.144 | 0.031 | 4.567 | <0.001 | 0.108 | 0.041 | 2.653 | 0.008 |
| Medical Science | 0.291 | 0.028 | 10.339 | <0.001 | 0.179 | 0.030 | 6.000 | <0.001 | 0.113 | 0.041 | 2.752 | 0.006 |
| Physics | 0.157 | 0.028 | 5.682 | <0.001 | 0.108 | 0.029 | 3.737 | <0.001 | 0.049 | 0.040 | 1.229 | 0.219 |
| Industry | -0.012 | 0.026 | -0.473 | 0.636 | 0.018 | 0.022 | 0.832 | 0.405 | -0.030 | 0.034 | -0.895 | 0.371 |
| University | 0.086 | 0.025 | 3.418 | 0.001 | 0.032 | 0.022 | 1.468 | 0.142 | 0.055 | 0.033 | 1.652 | 0.099 |

Notes: Standard errors clustered at respondent level. Reference categories are: Independent, White, Male, Economics, Government

Table 12: The Main results with Partisanship-adjusted Entropy Weights

|  | **(Non-partisan N=5020)** | | | | **(Partisan N=5030)** | | | | **(Difference N=10050)** | | | |
| --- | --- | --- | --- | --- | --- | --- | --- | --- | --- | --- | --- | --- |
|  | **Est.** | **SE** | **Stat.** | **P-Value** | **Est.** | **SE** | **Stat.** | **P-Value** | **Est.** | **SE** | **Stat.** | **P-Value** |
| Intercept | 0.295 | 0.025 | 12.036 | <0.001 | 0.461 | 0.043 | 10.613 | <0.001 | 0.461 | 0.043 | 10.619 | <0.001 |
| Democrat | NA | NA | NA | NA | -0.066 | 0.031 | -2.164 | 0.030 | NA | NA | NA | NA |
| Republican | NA | NA | NA | NA | -0.193 | 0.044 | -4.384 | <0.001 | NA | NA | NA | NA |
| Female | 0.012 | 0.016 | 0.765 | 0.444 | 0.021 | 0.016 | 1.327 | 0.184 | -0.009 | 0.022 | -0.411 | 0.681 |
| Asian American | 0.033 | 0.025 | 1.326 | 0.185 | 0.029 | 0.024 | 1.222 | 0.222 | 0.004 | 0.034 | 0.104 | 0.917 |
| Black | 0.013 | 0.022 | 0.591 | 0.554 | 0.039 | 0.023 | 1.732 | 0.083 | -0.026 | 0.032 | -0.829 | 0.407 |
| Hispanic | -0.014 | 0.022 | -0.630 | 0.529 | -0.011 | 0.024 | -0.481 | 0.631 | -0.002 | 0.032 | -0.068 | 0.946 |
| Env. Science | 0.210 | 0.024 | 8.799 | <0.001 | 0.148 | 0.025 | 5.952 | <0.001 | 0.062 | 0.034 | 1.800 | 0.072 |
| Medical Science | 0.303 | 0.025 | 12.154 | <0.001 | 0.181 | 0.024 | 7.421 | <0.001 | 0.123 | 0.035 | 3.520 | <0.001 |
| Physics | 0.163 | 0.024 | 6.681 | <0.001 | 0.120 | 0.023 | 5.244 | <0.001 | 0.043 | 0.033 | 1.292 | 0.196 |
| Industry | -0.009 | 0.022 | -0.405 | 0.686 | 0.046 | 0.020 | 2.320 | <0.001 | -0.055 | 0.030 | -1.852 | 0.064 |
| University | 0.080 | 0.022 | 3.736 | <0.001 | 0.033 | 0.021 | 1.614 | 0.107 | 0.047 | 0.030 | 1.594 | 0.111 |

Notes: Standard errors clustered at respondent level. Reference categories are: Independent, White, Male, Economics, Government

**References**

[1] Jens Hainmueller, Dominik Hangartner, and Teppei Yamamoto. Validating vignette and conjoint survey experiments against real-world behavior. Proceedings of the National Academy of Sciences, 112(8):2395–2400, 2015.

[2] Jens Hainmueller, Daniel J Hopkins, and Teppei Yamamoto. Causal inference in conjoint analysis: Understanding multidimensional choices via stated preference experiments. Political analysis, 22(1):1–30, 2014.

[3] Jens Hainmueller and Yiqing Xu. Ebalance: A stata package for entropy balancing. Journal of Statistical Software, 54(7), 2013.
