# Supplementary material for: Public perception of scientists: Experimental evidence on the role of sociodemographic, partisan, and professional characteristics
Source: PLoS One. 2023 Jul 7;18(7):e0287572. doi: 10.1371/journal.pone.0287572 (PMC10328236; doi:10.1371/journal.pone.0287572)
Supplement: Attachment — Submitted filename: Response to Reviewers.docx [file pone.0287572.s003.docx]

May 19, 2023

Dear Editors and Reviewers,

Thank you for the invitation to revise and resubmit our manuscript entitled “Public Perception of Scientists: Partisan and Non-partisan Thinking” (PONE-D-22-23245). We are grateful to both reviewers and editors for their detailed feedback and suggestions for minor revisions. We have edited the manuscript in regards to actionable suggestions and further comments. In line with the journal requirements, we have also reviewed our reference list to ensure that it is complete and correct.

As requested, we detail how we have addressed each of the comments and acted upon the suggestions. We have highlighted specific changes in the manuscript, using coloured (red) text.

**Reviewer 3:**

1. You did an adequate job responding to the suggestions of the reviewers. However, the quality of the figures should be addressed before publication.

Thanks for the feedback. Before resubmitting, we rechecked the figures and also used PLOS ONE’s suggested platform “PACE” to make sure the quality of the figures.

**Reviewer 4:**

1. First, I’m not sure the title is accurate – or at least, does not convey the main points of this paper. I wasn’t sure what “partisan and non-partisan thinking” meant when I started the paper, and I’m still not sure this well-captures the paper. It reduces the paper to the partisan cues/heuristics and I think doesn’t convey much of what the paper does. Perhaps “Public perception of scientists: partisan and non-partisan characteristics contributing to perceptions” or something similar?

Thanks for this feedback. Based on the suggestion, we revised the title to better convey the main points of the study. The updated title is “Public perception of scientists: Experimental evidence on the role of sociodemographic, partisan, and professional characteristics”

2. In some of the rationale and background supporting why the authors tested different characteristics of scientists, they conflate attributes of the scientists with those same attributes (or stereotypes related to those attributes) among the general public. This is most salient in the paragraph about race (111-122) where some of the justifications are about racialized stereotypes about scientists and some, such as racial differences in science literacy, are about the general public. I suggest authors edit this paragraph accordingly to be more clear that they are referring to racialized perceptions of scientists.

This paragraph has been edited to clarify that we are interested in racialised perceptions of scientists.

3. Also in the background (lines 168-176), is an alternative explanation for why some scientific fields might be regarded with less trust or credibility be how applied the science is into policy and politics? I think vaccination, infectious disease, climate change can also be characterized by how closely adjacent those fields are to relevant policy decisions that get embroiled in politics (mandates, restrictions, and taxes or other consumer restrictions) and not only how “permeated” they are by conspiratorial thinking.

Thank you for this insightful comment. We agree with the reviewer. A couple of sentences have been added to the manuscript (p. 8 lines 173-177) to incorporate this feedback into the background.

4. I know the interaction between partisanship and institution/place of work is only exploratory, but I still think it needs more explanation. For instance, the authors note that “Republicans and Democrats have contrasting views on institutions” (line 201) but do not explain what these views are.

This oversight has been corrected in the manuscript. As suggested by the reviewer, additional text has been added to (p. 10 lines 208-209) to flesh out those views.

5. The study was field in March of 2020. I know one of the previous reviewers asked the author to spell out how this timing might have been related to trust and the authors note that trust declines began later. However, there were other elements of partisan gaps in views about the pandemic that emerged as early as February and early March (seehttps://fivethirtyeight.com/features/how-concerned-are-americans-about-coronavirus-so-far/). I would like to see the authors engage a bit more about how the salience of the emerging pandemic might shape perhaps how respondents viewed reference to medical science in particular as a field, or to whether and how the stage of the pandemic could have affected survey response or other aspects of survey administration. (Please also note the actual dates that the study was fielded, not just “March of 2020”).

Thanks for this feedback. Based on the reviewer’s suggestion, we first clarified the actual data collection dates in the manuscript (p. 12 lines 249-250) and then how the partisan gap in the confidence in the U.S. health care system in dealing with the coronavirus may have an impact on respondents’ confidence in medical science as a field (p. 13 lines 260-268). More precisely, we highlighted that there was a partisan gap in the confidence in the U.S. health care system in dealing with the coronavirus. That is, in early March 2020, a vast majority (87%) of Republicans had confidence in the U.S. health care system to handle the response to the coronavirus, while a slight majority (53%) of Democrats had this positive view (Malloy and Schwartz 2020) (This new reference has been added to the list). This partisan heterogeneity in turn may have a differential impact on respondents’ confidence in medical science and potentially scientists in this scientific field. Hence, it is crucial to include measures of respondent’s partisanship in our study design, which can capture the main currents of difference between Republican and Democrat respondents.

- *Tim Malloy, Doug Schwartz. Biden crushes Sanders in democratic race, Quinnipiac University national poll finds; More disapprove of Trump’s response to coronavirus [Internet]. Quinnipiac University Poll; 2020. Available from: https://poll. qu. edu/national/release-detail.*

6. Like Reviewer 2, I too was surprised by the language of “Board of Scientific Councillors in your district.” This is not a concept I have ever heard of (as a U.S. citizen who studies science communication!) so I’m doubtful that most respondents had heard of this or knew what they were responding about. Further, the concept of “district” is not commonly used in the U.S. to refer to local government (as opposed to county, municipality, or town/city). District normally refers to schools, not other governmental entities. While I see the authors’ response to the initial reviewer who raised this, I would push back gently and suggest that there may be a limitation in the study design wherein there could be some unmeasured error in how respondents understood this concept that they were rating. Would suggest more acknowledgement or a footnote about this concern.

Thanks for raising this concern. We have added a couple of sentences (p. 28 lines 569-573) to clarify that the specific language used to define the scientific board in the task (i.e. Board of Scientific Councillors in their district) may have been misunderstood by the U.S. participants, because as the reviewer suggests, the concept is not widely known. We acknowledge that this limitation may lead to some unmeasured error in operationalising our dependent variables.

7. For the 3 key dependent variables, please state the measurement of the outcomes outright. Authors say they are measured on a Likert scale “where 7 is strongly trust and 1 is strongly mistrust” but this measurement does not make sense for the first DV (“where would you place your assessment”) nor the second “How much would you agree..”). It would be clearest to just note the response options for each item.

This oversight has been corrected in the manuscript (p. 16).

8. In the limitations, I would like to see the authors emphasize Reviewer 1’s very important concern about ecological validity more forcefully (lines 527-528). They acknowledge rather obliquely that the task is artificial, but I think they should acknowledge more explicitly that the task they are asking respondents to do (select a scientist to serve in a particular role) is something the general public will rarely if ever actually do. I think the trade-offs between the validity of this task and the causal inference of the design are justified, but it does bear more explicit discussion.

The discussion part (p.27-28 lines 565-569) has been revised to explicitly acknowledge that in our study, respondents take a decision task in which they are asked to elect a scientist, yet the general public is less likely to get involved in this type of elections in real-world context. The artificial setting therefore undermines the ecological validity of the findings.

***Minor points:***

Line 73 – “public preference for science is unequally distributed” – unequal distribution sounds like a moral claim, when I think the authors just mean “heterogeneous based on…”

Thanks. We agree with the reviewer. We changed it accordingly.

Line 77 – the reference to messages that scientists communicate is confusing, as this study does not address messaging at all. And yet in lines 77 through the Fauci example, the authors describe messages that trigger reactions based on audience predispositions. I would cut or edit this content as it made me think the paper was going to address communication and source x message interactions.

Based on the reviewer’s suggestion, we cut the content through Fauici’s example to avoid confusions (p. 4 line 79).

Line 95-96 – “a considerable fraction of stereotypes concerning sex roles conveys a denigrating message about women and often denies that they possess certain traits” – this is awkward and the subject-verb agreement is off (i.e., stereotypes convey messages; stereotypes deny that they possess); I would rephrase.

Thanks. We have rephrased this sentence to clarify the point (p. 5 lines-94-95).

Line 133 – “depending on whether they are state, industry, or academic” – should “state” be government, or is the reference to the U.S. states?

Yes, it should have been “government”. We have corrected this (p. 6 line 134).

Line 146 – “are open to being mistrusted from a different perspective.” This took me a while to understand. Perhaps another way to word this is that “scientists working for government…potentially face mistrust from a different perspective”?

We agree with the reviewer. We have revised the sentence (p. 7 lines 147-148).

Lines 165-167 – can authors clarify what they mean by autonomy-heteronomy?

Sure. It’s a Bourdieusian concept, which means that people differentiate between how knowledge relates to broader society, whether the audience is exclusively other knowledge producers (i.e. autonomous), or if economic and political authorities outside the knowledge domain are also the audience (i.e. heteronomous). However, we have decided to exclude this concept, since it creates ambiguity for the journal’s broad readership.

Figures – like Reviewer 1, I want to make sure that the figures have comprehensive notes attached. For instance, I know what the Partisan column is referring to in the first figure, and what the non-partisan column is, but what is “Nonpartisan-Partisan”? Double checking that there are complete notes and legends for all exhibits will be important, as this was hard for me to assess in how the figures appeared in the reviewer copy.

Thanks. We double-checked that notes are complete and clarified that the first panel (Nonpartisan) shows the results of the conjoint profiles without the partisan profiles. The second panel (Partisan) shows the results of the conjoint profiles with the partisan profiles. The third panel (Nonpartisan-Partisan) shows the differences between the partisan and nonpartisan profiles.

Overall, we are grateful to all reviewers and editorial team for their feedback and suggestions for improving this manuscript. We hope you agree that the revised manuscript is now much improved and suitable for publication.

Best regards,

The authors
